# Host–microbial co-metabolites: from biogenesis to immunomodulation and implications for health and disease

**DOI:** 10.3389/fimmu.2026.1775938

**Published:** 2026-07-03

**Authors:** Ying Wang, Liangliang Zhao, Ying Zou, Lili Nie

**Affiliations:** Department of Ophthalmology, the Second Hospital of Jilin University, Changchun, China

**Keywords:** bile acids, biomarker, co-metabolites, gut-brain axis, hippurate, immunomodulation, indoxyl sulfate, p-cresyl sulfate

## Abstract

Host–microbial co-metabolites are small bioactive molecules generated through obligatory sequential or complementary enzymatic transformations by both gut microbiota and host tissues, including secondary bile acids, trimethylamine *N*-oxide (TMAO), indoxyl sulfate, *p*-cresyl sulfate, phenylacetylglutamine, and hippurate. The dysregulation of this co-metabolic axis, often through gut microbial dysbiosis, contributes to chronic low-grade inflammation and has been implicated in inflammatory bowel disease, metabolic disorders, cardiovascular disease, kidney disease, neurological disorders, and cancer. This review synthesizes the definition, biogenesis, immunomodulatory mechanisms, disease relevance, and translational biomarker potential of strict host–microbial co-metabolites.

## 1 Introduction

The human body hosts a vast microbial ecosystem that participates actively in metabolic signaling with host tissues (1, 2). Host–microbial co-metabolites are distinct from metabolites produced independently by either partner: their final structure or systemic concentration depends on biochemical contributions from both microbial and host enzymes (3). This strict definition is important because many microbiota-associated molecules are microbial-derived bioactives rather than true co-metabolites. In this review, the term co-metabolite is reserved for molecules requiring host–microbial enzymatic collaboration, including secondary bile acids, trimethylamine *N*-oxide (TMAO), indoxyl sulfate, *p*-cresyl sulfate, phenylacetylglutamine, and hippurate.

The disruption of the host–microbial co-metabolite axis can shift host homeostasis toward chronic inflammation and metabolic dysfunction, contributing to obesity, cardiovascular disease, diabetes, kidney disease, and neuroinflammatory disorders (4). This review outlines the definition, biosynthetic pathways, immunomodulatory mechanisms, disease associations, and translational applications of strict host–microbial co-metabolites.

## 2 Host–microbial co-metabolites: definition, biogenesis, and mechanisms

Host–microbial co-metabolites are small-molecule metabolites whose final chemical structure or physiological concentration in the mammalian host is determined by sequential or complementary transformations carried out by both microbial and host enzymes on a shared substrate pool (5, 6). True co-metabolites therefore require obligatory biochemical collaboration: the removal of either microbial or host contributions abolishes the molecule, changes its final structure, or substantially alters its systemic concentration. This definition excludes microbial-only bioactives and host-only metabolites, even when they influence similar pathways.

Co-metabolites can be grouped into two broad biosynthetic classes: host-derived substrates modified by microbial enzymes, such as secondary and tertiary bile acids, and microbial metabolites further processed by host enzymes, such as TMAO, indoxyl sulfate, *p*-cresyl sulfate, phenylacetylglutamine, and hippurate. These molecules function as chemical messengers between gut microbial ecology and host physiology through receptor-mediated signaling, immune modulation, mitochondrial effects, and systemic metabolic regulation.

Within tryptophan-related metabolism, this review focuses only on strict host–microbial co-metabolites such as indoxyl sulfate, in which gut bacteria first generate indole, and host hepatic enzymes subsequently oxidize and sulfate it. Microbial-only or host-only products related to the same amino acid pathways are not treated as co-metabolites in this review.

Microbial glycyl radical enzymes, including choline TMA-lyase (CutC) and carnitine oxygenase (CntA), convert dietary phosphatidylcholine, choline, betaine, and l-carnitine into trimethylamine (TMA) (7). Host hepatic flavin-containing monooxygenases, particularly FMO3 and, to a lesser extent, FMO1, then oxidize TMA to TMAO (8). Related pathways include intermediates such as gamma-butyrobetaine and crotonobetaine, and some bacteria can convert carnitine directly to TMA (9). Aromatic amino acid pathways follow a similar co-metabolic logic: microbial enzymes convert phenylalanine to phenylacetic acid, which host liver and kidney enzymes conjugate with glutamine or glycine to form phenylacetylglutamine or phenylacetylglycine (10–12). The microbial conversion of tyrosine to *p*-cresol, followed by host sulfation or glucuronidation, yields *p*-cresyl sulfate or *p*-cresyl glucuronide (13–15).

Together, these molecules act as chemical messengers at the microbiota–host interface, influencing receptor-mediated signaling, immune modulation, mitochondrial function, and systemic metabolic regulation (**Table 1**).

**Table 1:** Combined concise summary of strict host–microbial co-metabolites, biosynthetic class, major mechanisms, and disease relevance.

Co-metabolite/class	Biogenesis criterion	Major mechanisms	Disease relevance
Secondary and tertiary bile acids (DCA, LCA, UDCA, and conjugated derivatives)	Host cholesterol-derived primary bile acids are deconjugated, dehydroxylated, epimerized, or transformed by gut microbes and then reabsorbed or re-conjugated by host tissues.	FXR/TGR5 signaling, Th17/Treg balance, epithelial barrier regulation, GLP-1 secretion, and mitochondrial and inflammatory signaling.	IBD, metabolic disease, NAFLD, CVD, autoimmune disease, neurological disorders, and cancer-related inflammation.
TMAO	Gut bacteria convert dietary choline/carnitine/betaine to TMA; host FMO enzymes oxidize TMA to TMAO.	NLRP3 inflammasome activation, HMGB1/TLR4/NF-κB signaling, altered cholesterol transport, platelet hyperreactivity, and mitochondrial/ER stress.	Atherosclerosis, thrombosis, CVD risk, metabolic syndrome, CKD-associated risk, neuroinflammation, and cancer-associated metabolic dysregulation.
Indoxyl sulfate	Gut bacteria convert tryptophan to indole; host CYP and SULT enzymes convert indole to indoxyl sulfate.	AhR signaling, oxidative stress, endothelial activation, MCP-1/E-selectin induction, tubular fibrosis, and p53/p21 senescence pathways.	CKD progression, uremic toxicity, vascular dysfunction, CAD, intestinal inflammation, and neurovascular injury.
p-Cresyl sulfate and p-cresyl glucuronide	Gut bacteria convert aromatic amino acids to p-cresol; host SULT or UGT enzymes generate sulfate or glucuronide conjugates.	EGFR/ANXA1/STAT3 signaling, MMP activation, BBB disruption or stabilization depending on conjugate, and vascular inflammation.	CKD-associated vascular and neurovascular dysfunction, BBB injury, and gut–brain–axis pathology.
Phenylacetylglutamine and phenylacetylglycine	Gut bacteria convert phenylalanine to phenylacetic acid; host liver/kidney conjugates it with glutamine or glycine.	Adrenergic receptor signaling, platelet activation, thrombotic potential, and aromatic amino acid co-metabolism.	ASCVD, thrombosis, metabolic risk, and urinary/metabolic biomarker applications.
Candidate 2MBC	Proposed pathway: microbial BCAA catabolism generates 2-methylbutyric acid, followed by host acylcarnitine conjugation; strict classification requires further validation.	Integrin α2β1 binding, cPLA2 activation, and enhanced platelet reactivity.	Acute coronary syndrome, ischemic stroke, obesity, diabetes, and thrombotic risk (candidate evidence).
Hippurate	Gut microbial metabolism generates benzoate and related aromatic intermediates; host liver/kidney conjugates benzoate with glycine.	Marker of microbial diversity, aromatic compound metabolism, glycine conjugation, glucose, and hepatic metabolic regulation.	Blood pressure, obesity, fatty liver disease, Crohn’s disease, metabolic health, and urinary biomarker applications.
Bile acid-derived immunoregulatory species (3-oxoLCA and isoalloLCA)	Microbial hydroxysteroid dehydrogenases transform secondary bile acids; host enterohepatic cycling maintains systemic exposure.	RORgamma-t inhibition, Treg induction, Th17 suppression, and mitochondrial ROS modulation.	Autoimmune inflammation, intestinal immune balance, and inflammatory disease models.

Microbial-only bioactives and host-only metabolites were omitted to maintain the review scope.

DCA, deoxycholic acid; LCA, lithocholic acid; UDCA, ursodeoxycholic acid; IBD, inflammatory bowel disease; CVD, cardiovascular disease; TMAO, trimethylamine *N*-oxide; TMA, trimethylamine; FMO, flavin-containing monooxygenase; ER, endoplasmic reticulum; CKD, chronic kidney disease; CAD, coronary artery disease; BBB, blood–brain barrier; ASCVD, atherosclerotic cardiovascular disease; ROS, reactive oxygen species; NAFLD, non-alcoholic fatty liver disease; BCAA, branched-chain amino acid(s).

### 2.1 Bile acids

**Figure 1:**
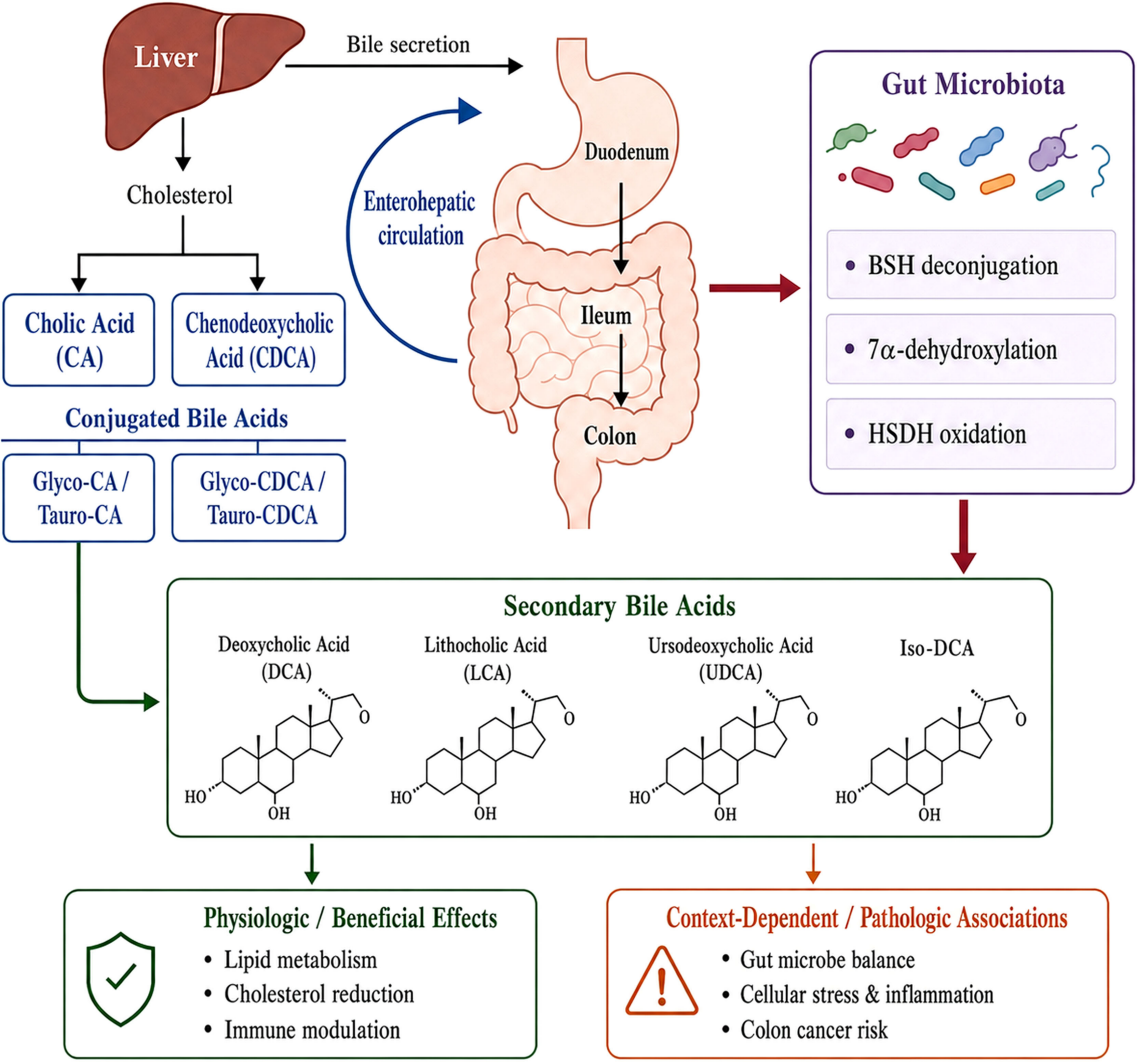
Bile acid co-metabolite signaling via FXR and TGR5. Primary bile acids synthesized in the liver are deconjugated and dehydroxylated by gut microbiota to form secondary bile acids (DCA, LCA, and UDCA) that activate nuclear receptor FXR and membrane receptor TGR5, regulating metabolic, immune, and inflammatory responses. FXR, farnesoid X receptor; TGR5, Takeda G protein-coupled receptor 5; DCA, deoxycholic acid; LCA, lithocholic acid; UDCA, ursodeoxycholic acid.

Bile acids are a prominent class of host–microbial co-metabolites (**Figure 1**) (16). The liver synthesizes primary bile acids from cholesterol, mainly cholic acid (CA) and chenodeoxycholic acid (CDCA), and conjugates them with glycine or taurine before biliary secretion (17–19). After release into the duodenum, most conjugated bile acids are reabsorbed in the ileum and returned to the liver through enterohepatic circulation, which preserves more than 95% of the bile acid pool (20). Gut bacteria expressing bile salt hydrolases deconjugate bile acids, while other taxa carry out dehydroxylation and epimerization reactions that generate secondary and tertiary bile acid species. Functional bile salt hydrolase activity is widespread across major gut microbial groups, including *Lactobacillus*, *Bifidobacterium*, *Clostridium*, and *Bacteroides* species (21–23).

### 2.2 Indoxyl sulfate

Indoxyl sulfate is a strict host–microbial co-metabolite derived from the sequential microbial and host metabolism of tryptophan-derived indole. Gut bacteria expressing tryptophanase convert tryptophan to indole, which crosses the intestinal epithelium and enters portal circulation. In the liver, cytochrome P450 enzymes oxidize indole to indoxyl, and sulfotransferases convert indoxyl to indoxyl sulfate. This host-conjugated molecule accumulates when renal clearance declines and acts through oxidative stress, aryl hydrocarbon receptor (Ahr) signaling, endothelial activation, and inflammatory pathways.

Indoxyl sulfate impairs vascular and renal function by increasing reactive oxygen species, promoting endothelial activation, inducing inflammatory chemokines, and accelerating tubular injury and fibrosis. Its concentration-dependent actions illustrate why host processing is central to the biological activity of many co-metabolites: the microbial precursor alone and the host-conjugated product can have distinct pharmacokinetics, tissue distribution, and toxicity profiles.

### 2.3 Phenylacetylglutamine and phenylacetylglycine

Phenylacetylglutamine is a nitrogen-rich co-metabolite that forms when phenylacetate (PAA), a product of gut microbes, is conjugated with glutamine in the liver and kidney (12). Protein-rich foods, particularly meat, are the principal dietary sources of phenylalanine (Phe), which is a precursor (12). Phenylacetylglutamine formation occurs in two main phases. Gut bacteria in the colon convert dietary Phe into PAA through two processes that depend on thiamine pyrophosphate (TPP). Microbial enzymes, including phenylalanine dehydrogenase (EC1.4.1.20) and aromatic amino acid aminotransferase (EC 2.6.1.57), convert Phe into phenylpyruvic acid (PPY) in one route (12). PPY is then converted into phenylacetyl-CoA by either oxidative decarboxylation by phenylpyruvate:ferredoxin oxidoreductase (PPFOR) or non-oxidative decarboxylation by phenylpyruvate decarboxylase (PPDC). *In vivo* and *in vitro* studies have indicated that both oxidative and non-oxidative decarboxylation play roles in microbial PAA synthesis (12). After formation, PAA enters the portal circulation and reaches the liver and kidneys (12). There, it is conjugated with glutamine or glycine by phenylacetyltransferase and glycine *N*-phenylacetyltransferase to make phenylacetylglutamine (PAGln) and phenylacetylglycine, respectively (12). PAGln predominates in humans, whereas phenylacetylglycine is more common in rodents (24–26).

### 2.4 Trimethylamine *N*-oxide

TMAO is a strict host–microbial co-metabolite formed when gut bacteria convert dietary choline, l-carnitine, betaine, and related precursors into TMA, which is then oxidized mainly by host hepatic FMO3 to TMAO (7, 8, 27). Microbial CutC/D and CntA/B pathways differ across taxa, and host FMO activity determines the final systemic TMAO pool. This two-step host–microbial pathway explains why TMAO links diet, microbial ecology, and host cardiometabolic risk.

### 2.5 Hippurate

Hippurate is a common host–microbial co-metabolite in human urine and has been associated with metabolic health, including inverse correlations with blood pressure, fatty liver disease, visceral adiposity, and Crohn’s disease (28, 29). Its microbial precursor, benzoate, is absorbed and conjugated with glycine in the liver and kidney to form hippurate (30). Experimental and human studies have suggested that hippurate levels reflect both host factors and gut microbiota diversity (31, 32).

Hippurate has shown inverse associations with blood pressure, obesity, and hepatic steatosis, supporting a diet-dependent link between microbial benzoate metabolism and host metabolic health (29, 33). In mouse models, urinary hippurate measured before high-fat diet exposure has predicted subsequent obesity risk, suggesting that microbial functional diversity can influence disease susceptibility independently of host genetics (34). Experimental studies have also suggested that hippurate and benzoate can improve glucose handling and insulin secretion, while hippurate can increase β-cell mass and reduce liver fibrosis (35). These findings support hippurate as both a marker of gut microbial metabolic capacity and a potential mediator of host metabolic resilience.

## 3 Co-metabolites in immunomodulation and disease mechanisms

**Figure 2:**
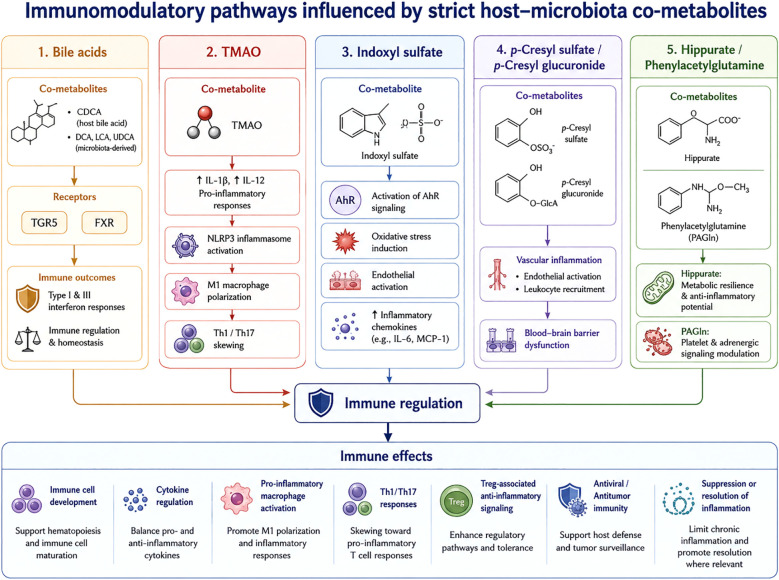
Immunomodulatory pathways influenced by strict host -microbial co-metabolites. This schematic illustrates immune regulatory mechanisms associated with strict host -microbial co-metabolites, including bile acid derivatives, TMAO, indoxyl sulfate, *p*-cresyl sulfate/*p*-cresyl glucuronide, hippurate, and phenylacetylglutamine. These metabolites influence immune regulation through receptor-mediated signaling, inflammasome activation, oxidative stress, endothelial activation, cytokine regulation, macrophage polarization, Th1/Th17 responses, Treg-associated anti-inflammatory pathways, antiviral and antitumor immunity, and suppression or resolution of inflammation, where relevant. TMAO, trimethylamine *N*-oxide.

Host–microbial co-metabolites regulate immunity by linking microbial activity with mucosal and systemic immune responses (**Figure 2**). Their effects are dose- and tissue-specific, influencing immune cell differentiation, epithelial barrier integrity, and inflammatory resolution. Bile acids can modulate receptor-mediated signaling and reshape microbial communities through direct antimicrobial activity and the regulation of host innate immunity (36). Signaling through the bile acid receptor TGR5 can enhance antiviral and antitumor immunity by increasing type I interferon (IFN-I) responses; for example, CDCA has been shown to increase serum IFN-β levels (37). Microbially modified bile acids such as lithocholic acid (LCA) and deoxycholic acid (DCA) can also induce type III interferon responses in the small intestine, while DCA generated by *Clostridium scindens* can restore plasmacytoid dendritic cell and MyD88-dependent IFN-I responses (38, 39). IFNs are central to cancer immunosurveillance because they can activate IFN-stimulated genes and enhance responses to selected anticancer therapies (40).

TMAO can promote inflammatory signaling through NLRP3 inflammasome activation and induction of IFN-stimulated genes. These pathways favor an M1-like macrophage phenotype characterized by pro-inflammatory cytokines, co-stimulatory molecules, and support for Th1/Th17 differentiation (41, 42). TMAO can also shift T-cell transcriptional programs away from regulatory phenotypes and toward stronger CD8 effector activity through regulators such as RICTOR and FOXP3 (42).

Host-conjugated indole co-metabolites, particularly indoxyl sulfate, modulate immune and inflammatory responses through AhR signaling, oxidative stress, endothelial activation, and inflammasome-related pathways. These actions are context- and concentration-dependent: low-level AhR signaling can support barrier homeostasis, whereas the accumulation of indoxyl sulfate in kidney disease promotes inflammation, vascular dysfunction, and tissue injury.

Emerging evidence also links host–microbial co-metabolites to cellular ageing processes, particularly mitophagy and the senescence-associated secretory phenotype (SASP). Secondary bile acids, including DCA, LCA, TMAO, and indoxyl sulfate, can influence mitochondrial quality control and inflammatory senescence pathways in intestinal, vascular, and renal cells. These findings suggest that the co-metabolite–mitophagy–SASP axis can be a therapeutic target in age-related diseases.

These immunometabolic mechanisms help explain why strict host–microbial co-metabolites are increasingly implicated in cardiovascular, renal, neurological, metabolic, autoimmune, and oncological disease contexts (**Table 1**). 

### 3.1 Cardiovascular disease

**Figure 3:**
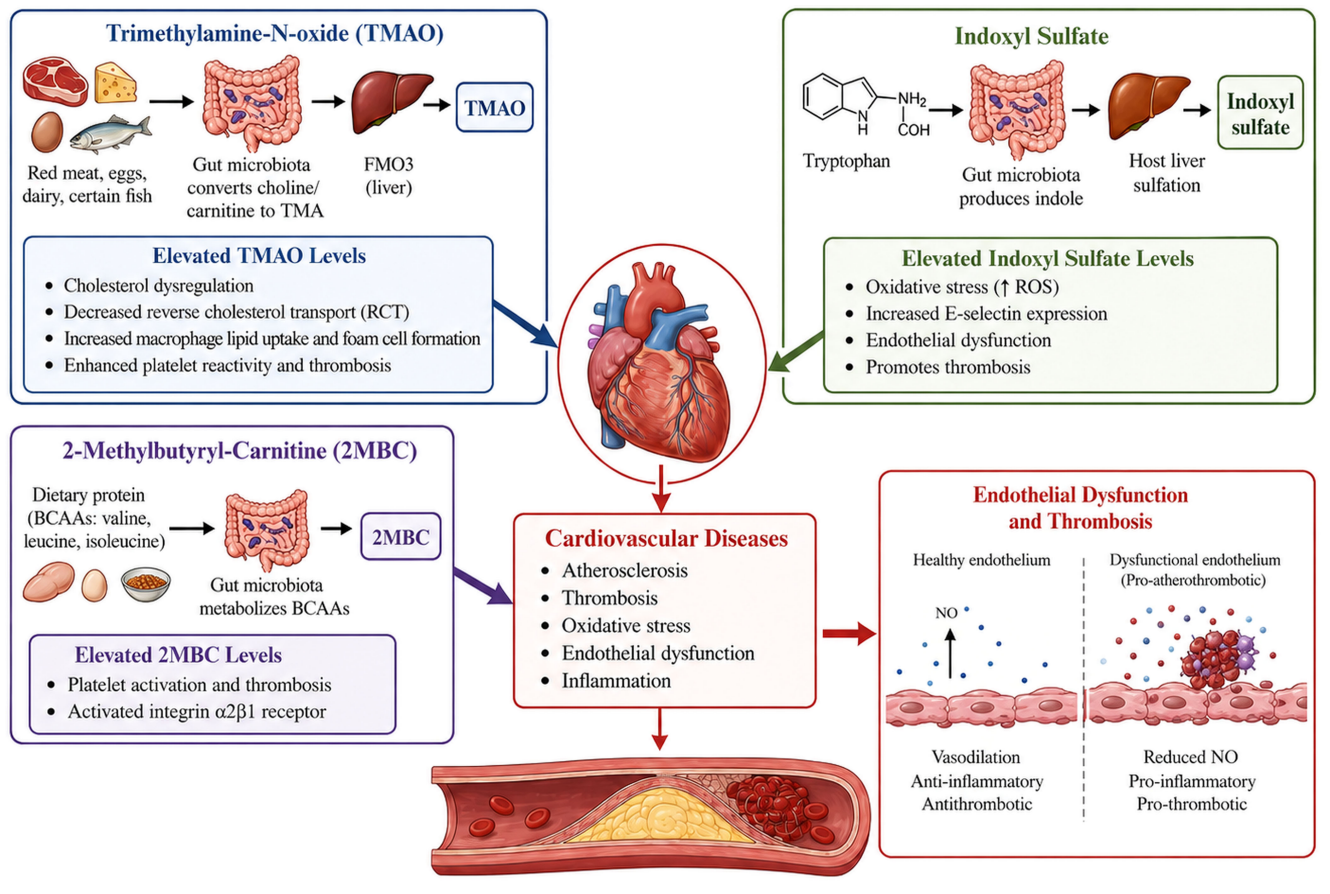
Host–microbial co-metabolites in cardiovascular disease. **(A)** TMAO impairs reverse cholesterol transport via HDL and promotes foam cell formation and platelet hyperreactivity. **(B)** Indoxyl sulfate induces endothelial ROS production and smooth muscle cell proliferation, accelerating atherosclerosis. **(C)** Phenylacetylglutamine activates α2A/α2B adrenergic receptors, enhancing platelet aggregation and thrombotic risk. **(D)** Hippurate inversely correlates with blood pressure and hepatic steatosis. HDL, high-density lipoprotein; ROS, reactive oxygen species; TMAO, trimethylamine *N*-oxide.

Co-metabolites generated through gut microbial and host enzymatic pathways have been strongly linked to cardiovascular disease (**Figure 3**) (43, 44). TMAO is associated with cardiovascular risk and mortality and can contribute mechanistically through altered cholesterol handling, endothelial dysfunction, inflammation, and platelet hyperreactivity (45–48). TMAO can impair reverse cholesterol transport, promote macrophage foam cell formation, activate inflammatory pathways such as HMGB1/TLR4/NF-κB, increase endothelial adhesion molecules, and enhance platelet calcium signaling.

2-Methylbutyryl-carnitine (2MBC) has recently been proposed as a candidate gut microbiota-linked co-metabolite that can increase thrombotic risk by enhancing platelet reactivity (49). Mechanistically, 2MBC has been reported to bind integrin α2β1 and augment cPLA2-dependent platelet activation. Human data linking elevated plasma 2MBC with acute coronary syndrome, ischemic stroke, obesity, and diabetes support its potential relevance as a thrombometabolic risk marker, although its classification as a strict co-metabolite and its clinical utility require independent validation.

Indoxyl sulfate has been investigated in coronary artery disease because of its capacity to induce oxidative stress and endothelial activation (50). In endothelial cell models, clinically relevant concentrations have increased reactive oxygen species, E-selectin expression, and inflammatory signaling, although reported effects vary across experimental systems and concentrations. These findings support a plausible link between microbial indole production, host sulfation, vascular inflammation, and atherosclerotic risk.

### 3.2 Kidney disease

Indoxyl sulfate is one of the best-characterized co-metabolites in chronic kidney disease (CKD). In healthy individuals, circulating concentrations remain relatively low, and most indoxyl sulfate is protein-bound; as renal function declines, free and total concentrations increase because tubular secretion and glomerular clearance become insufficient (51). The accumulation of indoxyl sulfate is associated with CKD progression and has been evaluated as a prognostic marker in dialysis and advanced kidney disease cohorts.

Mechanistically, indoxyl sulfate promotes renal and systemic injury by increasing oxidative stress, mitochondrial reactive oxygen species (ROS) production, inflammatory gene expression, tubular cell dysfunction, and fibrotic remodeling (51). Experimental studies have also suggested that indoxyl sulfate can disrupt intestinal homeostasis and activate inflammatory responses in epithelial cells and macrophages, linking renal failure, gut barrier dysfunction, and systemic inflammation (52–54).

### 3.3 Nervous system diseases

**Figure 4:**
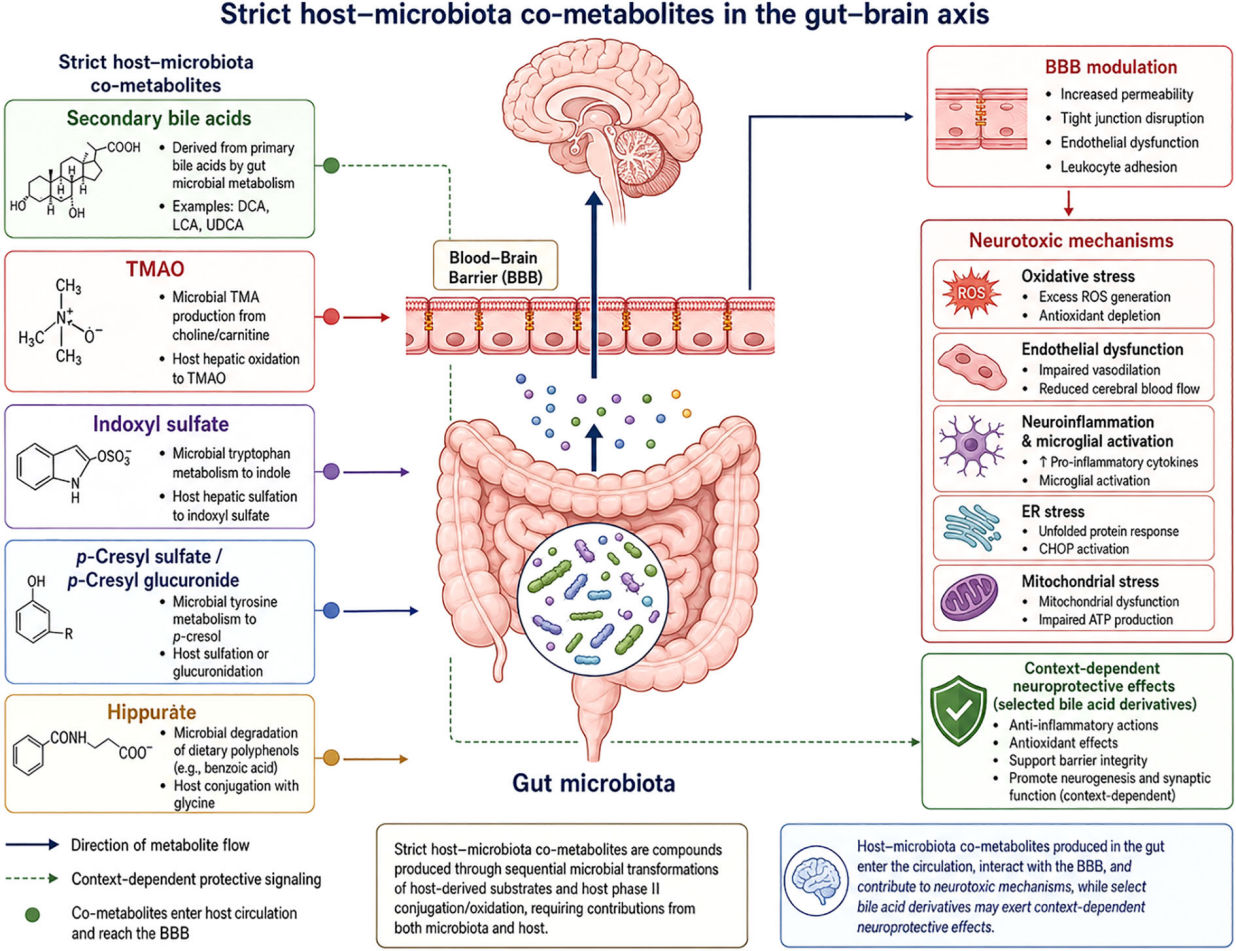
Strict host–microbial co-metabolites in the gut–brain axis. This schematic summarizes major strict host–microbial co-metabolites that can influence the gut–brain axis through systemic circulation, blood–brain barrier modulation, endothelial dysfunction, oxidative stress, neuroinflammation, microglial activation, endoplasmic reticulum stress, and mitochondrial stress. The figure includes only metabolites requiring both microbial and host biochemical contributions, including secondary bile acids, trimethylamine *N*-oxide (TMAO), indoxyl sulfate, *p*-cresyl sulfate/*p*-cresyl glucuronide, and hippurate. Secondary bile acids can also exert context-dependent neuroprotective effects through anti-inflammatory, antioxidant, and barrier-supporting mechanisms.

Strict host–microbial co-metabolites, including bile acid derivatives, TMAO, indoxyl sulfate, *p*-cresyl sulfate, and *p*-cresyl glucuronide, influence the microbiota–gut–brain axis through systemic inflammation, blood–brain barrier function, mitochondrial stress, and neuroimmune signaling (**Figure 4**). Microbial-only neuroactive bioactives are not reviewed here as co-metabolites, unless they undergo obligatory host transformation.

Bile acid profiles have been linked to cognitive decline, stroke outcomes, and neuroinflammatory disease. Altered ratios of bacterially modified bile acids have been associated with cognitive deterioration, whereas ursodeoxycholic acid and related bile acid derivatives can exert neuroprotective effects through reduced microglial activation, NLRP3 modulation, and mitochondrial protection (55–58).

TMAO and indoxyl sulfate can affect brain function indirectly through vascular inflammation, endothelial dysfunction, endoplasmic reticulum (ER) stress, oxidative signaling, and autonomic or neuroimmune pathways (59, 60). Similarly, *p*-cresyl sulfate can compromise neurovascular integrity by engaging epidermal growth factor receptor–matrix metalloproteinase (EGFR–MMP) signaling at the blood–brain barrier (BBB), whereas *p*-cresyl glucuronide can exert distinct BBB-stabilizing effects (61b; 62). These examples highlight that closely related conjugated co-metabolites can have divergent biological effects.

### 3.4 Metabolic diseases

**Figure 5:**
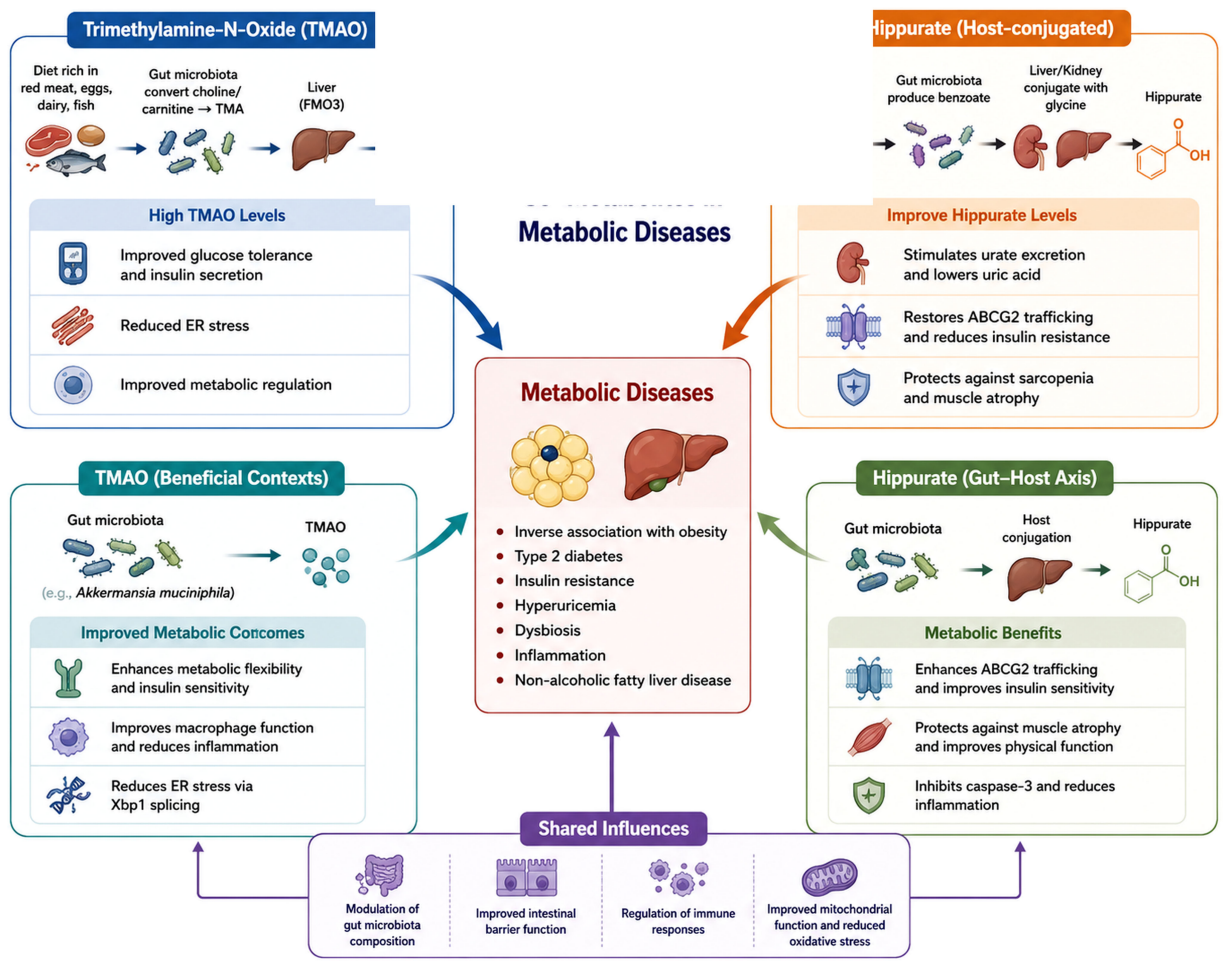
Host–microbial co-metabolites in metabolic diseases. **(A)** TMAO and bile acid dysmetabolism associate with obesity, insulin resistance, and hepatic steatosis. **(B)** Indoxyl sulfate and pCS accelerate CKD progression through tubular oxidative stress and NLRP3 inflammasome activation. **(C)** Secondary bile acid signaling via FXR/TGR5 regulates GLP-1 secretion and glucose homeostasis in type 2 diabetes. **(D)** Hippurate and PAGln serve as urinary biomarkers of gut microbial metabolic capacity and dietary fiber intake. CKD, chronic kidney disease; GLP-1, glucagon-like peptide-1; pCS, *p*-cresyl sulfate; TMAO, trimethylamine *N*-oxide; PAGln, phenylacetylglutamine.

Host–microbial co-metabolites also contribute to metabolic disease risk and can serve as early markers of metabolic resilience (**Figure 5**). Baseline urinary or circulating levels of TMAO and hippurate have been associated with obesity-related outcomes, insulin resistance, hepatic steatosis, and broader metabolic health signatures (31, 34, 63). The direction of association can vary by the route of exposure, host metabolic state, and microbial composition, underscoring the context-specific biology of these molecules.

Hippurate is increasingly viewed as both a marker of microbial functional diversity and a potential mediator of metabolic health. In hyperuricemia models, increased microbial production of hippurate has been linked to improved urate handling through ABCG2-related mechanisms, supporting the concept that selected co-metabolite pathways can be therapeutically modifiable (35, 64, 65).

### 3.5 Autoimmune diseases

Bile acid co-metabolites can regulate autoimmune inflammation through FXR/TGR5 signaling, intestinal barrier effects, and Th17/Treg balance. In rheumatoid arthritis models, cholic acid and microbiota-driven bile acid remodeling have been linked to reduced inflammatory responses and the preservation of joint integrity (66, 67).

In multiple sclerosis and experimental autoimmune encephalomyelitis, altered bile acid metabolism has been associated with reduced levels of secondary bile acids, including deoxycholic acid, and increased Th17 activity (68, 69). The restoration of immune regulatory bile acid pathways can therefore represent a mechanistic link between gut microbial dysbiosis and systemic autoimmune inflammation.

### 3.6 Cancer

In cancer, strict host–microbial co-metabolites can influence tumor biology through bile acid signaling, vascular inflammation, platelet activation, and immune modulation. Secondary bile acids have been linked to pro-carcinogenic signaling in gastrointestinal and hepatobiliary contexts, whereas TMAO and phenylacetylglutamine have emerging associations with inflammatory and thrombotic pathways that can shape the tumor microenvironment.

Bile acids have been associated with pancreatic and gastrointestinal cancer biology through COX-2/prostaglandin signaling, STAT3/MAPK/EGFR activation, mucin regulation, and autophagy-related pathways (70–73). These mechanisms suggest that microbial remodeling of bile acid pools can alter epithelial stress responses and tumor-promoting inflammation.

TMAO has been less extensively studied in cancer than in cardiovascular disease, but available evidence links it to NLRP3 inflammasome activation, NF-κB signaling, oxidative stress, and inflammatory pathways relevant to tumor progression (74–78). Further work is needed to determine whether TMAO acts as a causal driver, a biomarker of dysbiosis, or both.

## 4 Host–microbial co-metabolites as health and disease markers

Strict host–microbial co-metabolites are increasingly being evaluated as biomarkers because they integrate diet, microbial functional capacity, host conjugation pathways, renal or hepatic clearance, and inflammatory status. Among the most studied candidates are TMAO and phenylacetylglutamine for cardiovascular risk, indoxyl sulfate and *p*-cresyl sulfate for kidney disease and vascular injury, and hippurate for microbial diversity and metabolic health.

TMAO and phenylacetylglutamine have been associated with cardiometabolic and thrombotic risk, although their clinical interpretation requires attention to diet, renal function, microbial composition, and host enzyme activity. Similarly, indoxyl sulfate and *p*-cresyl sulfate are clinically relevant because their accumulation reflects both microbial precursor generation and impaired host clearance, making them useful candidates for risk stratification in chronic kidney disease and related vascular complications.

Hippurate is a useful example of a potentially protective co-metabolic marker. Higher levels of urinary or circulating hippurate have been associated with greater microbial gene richness, healthier metabolic profiles, and improved glucose handling in selected human and preclinical studies. These findings support hippurate as both a marker of microbial functional diversity and a possible mediator of metabolic resilience.

Future biomarker development should combine targeted metabolomics with metagenomics, metatranscriptomics, stable isotope tracing, and clinical phenotyping. Such integrated approaches are needed to distinguish true host–microbial co-metabolites from microbial-only bioactives, identify the enzymatic sources of circulating metabolites, and determine whether these molecules are causal mediators, disease markers, or modifiable therapeutic targets.

## 5 Concluding remarks and future directions

The dynamics of host–microbial co-metabolism represent an important framework for understanding human physiology. Bile acid derivatives, TMAO, indoxyl sulfate, *p*-cresyl sulfate, phenylacetylglutamine, and hippurate exemplify how obligatory host–microbial biochemical collaboration can regulate immunity, metabolism, vascular function, kidney injury, and neuroinflammation. Applying a strict definition distinguishes true co-metabolites from microbial-derived bioactives and improves the conceptual scope of this review.

New technologies are improving the ability to define where host–microbial co-metabolites originate, how they move across tissues, and how they influence physiology. High-resolution metabolomics can profile small-molecule intermediates, whereas shotgun metagenomics and metatranscriptomics clarify the genetic potential and transcriptional dynamics of microbial populations involved in co-metabolism. Stable isotope tracing, including ^13^C, ^15^N, and deuterium labeling, is essential for distinguishing microbial from host contributions and for quantifying co-metabolic flux.

In clinical practice, strict host–microbial co-metabolites are emerging as biomarkers and therapeutic targets. Plasma or urinary levels of TMAO, phenylacetylglutamine, indoxyl sulfate, *p*-cresyl sulfate, and hippurate are increasingly investigated for cardiovascular, metabolic, renal, and neurological risk stratification. Modulating these pathways through diet, precision probiotics, enzyme inhibition, or receptor-directed therapy can provide clinically useful strategies, but validation in prospective cohorts remains essential.

Future work should integrate metabolomics, microbial functional profiling, and clinical phenotyping to identify co-metabolites that can serve as biomarkers of disease progression, treatment response, or dietary intervention. Such approaches can help translate host–microbial co-metabolism into diagnostic tools and targeted therapies.
